# Lymph node ratio predicts efficacy of postoperative radiation therapy in nonmetastatic Merkel cell carcinoma: A population‐based analysis

**DOI:** 10.1002/cam4.4773

**Published:** 2022-04-29

**Authors:** Giuseppe Lamberti, Elisa Andrini, Giambattista Siepe, Cristina Mosconi, Valentina Ambrosini, Claudio Ricci, Paola Valeria Marchese, Gianluca Ricco, Riccardo Casadei, Davide Campana

**Affiliations:** ^1^ Department of Experimental Diagnostic and Specialized Medicine (DIMES), Alma Mater Studiorum University of Bologna Bologna Italy; ^2^ Division of Medical Oncology, IRCCS Azienda Ospedaliero‐Universitaria di Bologna Bologna Italy; ^3^ NET Team Bologna ‐ ENETS Center of Excellence Bologna Italy; ^4^ Radiation Oncology, IRCCS Azienda Ospedaliero‐Universitaria di Bologna Bologna Italy; ^5^ Department of Radiology, IRCCS Azienda Ospedaliero‐Universitaria di Bologna Bologna Italy; ^6^ IRCCS Azienda Ospedaliero‐Universitaria di Bologna Bologna Italy; ^7^ Department of Internal Medicine and Surgery (DIMEC), Alma Mater Studiorum University of Bologna Bologna Italy

**Keywords:** LNR, lymph node ratio, Merkel cell carcinoma, radiation therapy, Radoitherapy, SEER

## Abstract

**Background:**

After radical resection of a nonmetastatic Merkel cell carcinoma (M0 MCC), postoperative radiation therapy (RT) is recommended as it improves survival. However, the role of RT in specific subgroups of M0 MCC is unclear. We sought to identify whether there is a differential survival benefit from RT in specific M0 MCC patient subgroups.

**Methods:**

M0 MCC patients from the Surveillance, Epidemiology, and End Results (SEER) database registry were collected. The best prognostic age, tumor size, and lymph node ratio (LNR, ratio between positive lymph nodes and resected lymph nodes) cutoffs were calculated. The primary endpoint was overall survival (OS).

**Results:**

A total of 5644 M0 MCC patients (median age 77 years, 62% male) were included: 4022 (71%) node‐negative (N0) and 1551 (28%) node‐positive (N+). Overall, 2682 patients (48%) received RT. Age > 76.5 years, tumor size >13.5 mm, and LNR >0.215 were associated with worse OS. RT was associated with longer OS in the M0 MCC, N0, and N+ group and independently associated with a 25%, 27%, and 26% reduction in the risk for death, respectively. RT benefit on survival was increased in tumor size >13.5 mm in the N0 group and LNR >0.215 in the N+ group. No OS benefit from RT was observed in T4 tumors (N0 and N+ groups).

**Conclusions:**

RT was associated with improved survival in M0 MCC, irrespective of the nodal status. LNR >0.215 is a useful prognostic factor for clinical decision‐making and for stratification and interpretation of clinical trials.

## Graphical abstract

There are no factors to select patients with non‐metastatic Merkel cell carcinoma for radiotherapy. Increased benefit from radiotherapy in patients with tumors >13.5 mm (node‐negative) and in those with lymph‐node ratio >0.215 (node‐positive) was observed, but not in T4 tumors (either group).

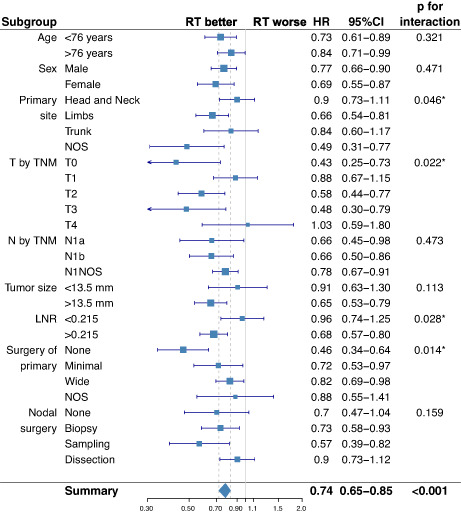

## 1 BACKGROUND

Merkel cell carcinoma (MCC) is a rare and aggressive neuroendocrine neoplasia of the skin,^1^ whose incidence is rising^2^, ^3^ and whose mortality is the highest among skin cancers, including melanoma.^4^ In addition, given its nonspecific presentation, MCC is often diagnosed at an advanced stage, with consequent poor prognosis.^5^, ^6^ Recently, the introduction of immunotherapy with immune checkpoint inhibitors for the treatment of advanced MCC significantly improved the survival outcomes of these patients,^7^ while a multimodal approach that includes a combination of excision of the primary lesion, sentinel lymph node biopsy (SLNB), nodal dissection, and postoperative radiation therapy (RT) is usually required for the management of nonmetastatic MCC.^8^, ^9^

Postoperative RT has been associated with improved local control in patients with nonmetastatic MCC, while its impact on survival is controversial.^10^, ^11^, ^12^, ^13^, ^14^, ^15^, ^16^, ^17^, ^18^, ^19^, ^20^ In addition, whether there are subgroups of patients deriving differential benefits from RT is currently unknown. Nevertheless, RT is recommended after surgery despite conflicting results regarding its effect on survival, irrespective of nodal involvement,^8^, ^9^ but there is no evidence of differential survival benefits from RT to help select patients for adjuvant treatment, such as RT, after radical resection of a nonmetastatic MCC.

To identify subgroups of MCC patients who may benefit most from RT, we queried the Surveillance, Epidemiology, and End Results (SEER) database and analyzed the factors associated with survival in specific subgroups of nonmetastatic MCC (M0 MCC) patients in an unbiased way, with a focus on the lymph node ratio (LNR).

## 2 METHODS

We sought to evaluate in an unbiased way the efficacy in terms of overall survival (OS) of RT in the treatment of localized MCC in a population‐based analysis. The SEER registry was interrogated using the SEER*stat software (https://seer.cancer.gov) to include all patients with a diagnosis of nonmetastatic MCC and no distant metastases. Records were selected by histology according to ICD‐O‐3 diagnosis code 8247 from the “Incidence ‐ SEER 18 Regs Research Data + Hurricane Katrina Impacted Louisiana Cases (with additional treatment fields), Nov 2018 Sub (1975‐2016 varying)” based on the November 2018 submission database, and clinical and pathology data were collected. The primary sites were grouped in head and neck, limbs (including upper limb and shoulder, lower limb, and hip), trunk, and other/not otherwise specified (other/NOS). Resection of the primary tumor was classified as “none” if no surgery was performed on the primary tumor, “minimal” (including excisional biopsy, local excision, laser ablation, electrocauterization, lumpectomy, Mohs resection with ≤1 cm margin), or “wide” (including wide excision, amputation, biopsy followed by wide excision and Mohs resection with >1 cm margins). Lymph node‐directed surgery was classified as “none” if no surgery was performed on lymph nodes, “biopsy” (including nodal biopsy and sentinel lymph node biopsy), “sampling” (including excision of ≤3 lymph nodes), or “dissection” (including lymphadenectomy and excision of ≥4 lymph nodes). The LNR was calculated as the ratio between the number of positive lymph nodes and the total number of analyzed lymph nodes in all patients with at least one positive lymph node. Permission to access the SEER database was granted on 19/03/2020 with authorization number 21495‐Nov2018.

### 2.1 Statistical analysis

The primary endpoint of the analysis is OS, defined as the time from diagnosis to death by any cause, estimated by the Kaplan–Meier method, reported in months (95% confidence interval [CI]). OS was chosen over cancer‐specific survival to capture detrimental effects of treatments on survival, since patients with MCC are characterized by older age and comorbidities, and to avoid the introduction of biases in the assessment of the cause of death. Results were compared with the log‐rank method. Predictive risk factors for OS were analyzed by univariate and multivariate analyses using the Cox proportional hazards method and expressed as hazard ratios (HR) [95% CI]. The multivariate model was fitted using the backward stepwise method after including all variables. The area under the receiver‐operating characteristic (ROC) curve was evaluated to determine the accuracy of age, tumor size in millimeters (mm), and LNR in predicting vital status at 5 years from diagnosis. The best prognostic cutoff value was estimated by using Youden's statistics. Subgroup analyses were represented by a Forest plot. The *p* value was considered significant when <0.05. The statistical analysis was carried out using IBM—SPSS Statistics v. 22 and R Statistical package version 3.6.1 software.

## 3 RESULTS

### 3.1 Overall patient characteristics

Records from 9773 patients with MCC were extracted from the SEER database and 5644 patients with a diagnosis of nonmetastatic MCC (M0 MCC) were finally included (Figure S1). Age > 76.5 years, tumor size >13.5 mm, and LNR >0.215 were significantly associated with the survival status 5 years after diagnosis (Figure S2). Patient characteristics of the M0, N0 (without nodal involvement), and N+ groups (with lymph node involvement) are summarized in Table 1. Notably, no imbalance was observed in RT delivery according to LNR in the N+ group (Table S1).

**TABLE 1:** Patient characteristics by Merkel cell carcinoma patient cohort

	MCC cohort	M0		N0		N+	
Variable		*N*	*(%)*	*N*	*(%)*	*N*	*(%)*
*N*		5644		4022	(71.3%)	1551	(27.5%)
Age	Median (range)	77	(12–106)	78	(12–106)	75	(31–100)
	>76.5 years	2953	(52.3%)	2225	(39.5%)	674	(44.7%)
Sex	Male	3518	(62.3%)	2434	(60.5%)	1029	(66.3%)
	Female	2126	(37.7%)	1588	(39.5%)	522	(33.7%)
Primary site	Head and Neck	2464	(43.7%)	1887	(46.9%)	547	35.3%
	Limbs	2415	(42.8%)	1760	(43.8%)	625	(40.3%)
	Trunk	573	(10.1%)	354	(8.8%)	212	(13.7%)
	NOS	192	(3.4%)	21	(0.5%)	167	(10.8%)
Stage at diagnosis	I	1845	(42.7%)	1845	(45.9%)	–	
	II	857	(19.8%)	857	(54.1%)	–	
	III	1622	(37.5%)	–		1551	(100%)
	NA	1320		1320			
T by TNM	T0	133	(3.5%)	‐		130	(8.4%)
	T1	2261	(59.4%)	1827	(67.6%)	416	(26.8%)
	T2	996	(26.2%)	652	(24.1%)	316	(20.4%)
	T3	234	(6.1%)	124	(3.1%)	96	(6.2%)
	T4	182	(4.8%)	99	(3.7%)	75	(4.8%)
	NA	1838		1320		518	
Tumor size	Median, *mm* (range)	17	1–500	15	1–500	21	1–180
	≤13.5 mm	1445	(40.0%)	1191	(44.8%)	242	(27.1%)
	>13.5 mm	2163	(60.0%)	1468	(55.2%)	650	(72.9%)
	NA	2036		1363		659	
N by TNM	N0	4022	(71.3%)	4022	(100%)	–	
	N1a	237	(4.2%)	–		237	(15.3%)
	N1b	402	(7.1%)	–		402	(25.9%)
	N1 NOS	912	(16.1%)	–		912	(58.8%)
	N2	71	(1.3%)	–		‐	
LNR	≤0.215	469	(34.8%)	–		455	(34.7%)
	>0.215	878	(65.2%)	–		855	(65.3%)
	NA	4297		–		241	
Surgery of primary	None	502	(8.9%)	214	(5.3%)	276	(17.8%)
	Minimal	1365	(24.2%)	193	(27.2%)	261	(16.8%)
	Wide	3639	(64.5%)	2669	(66.4%)	922	(59.5%)
	NOS	138	(2.4%)	46	(1.1%)	92	(5.9%)
Nodal surgery	None	2207	(40.4%)	2058	(52.2%)	131	(9.0%)
	Biopsy	1838	(33.7%)	1349	(33.5%)	470	(32.5%)
	Sampling	424	(7.8%)	212	(5.4%)	202	(14.0%)
	Dissection	988	(18.1%)	322	(8.2%)	644	(44.5%)
	NA	187		81		104	
Radiation therapy	Yes	2682	(47.5%)	1637	(40.7%)	998	(64.3%)
	No	2962	(52.5%)	2385	(59.3%)	553	(35.7%)

Abbreviations: LNR, lymph node ratio; NA, not available.

### 3.2 Prognostic factors in M0 MCC patients

After a median follow‐up of 78 months (95% CI 76–82), the median OS was 55 months (95% CI 51–59), with a 5‐year OS rate of 48%. Comparisons of survival by key prognostic groups are reported in Table S2 and Figure S3. Patients who underwent perioperative RT had longer OS compared with those who did not (67 months [95% CI 60–74] vs 48 months [95% CI 44–52], respectively; *p* < 0.001, Figure S4A).

**FIGURE 1:**
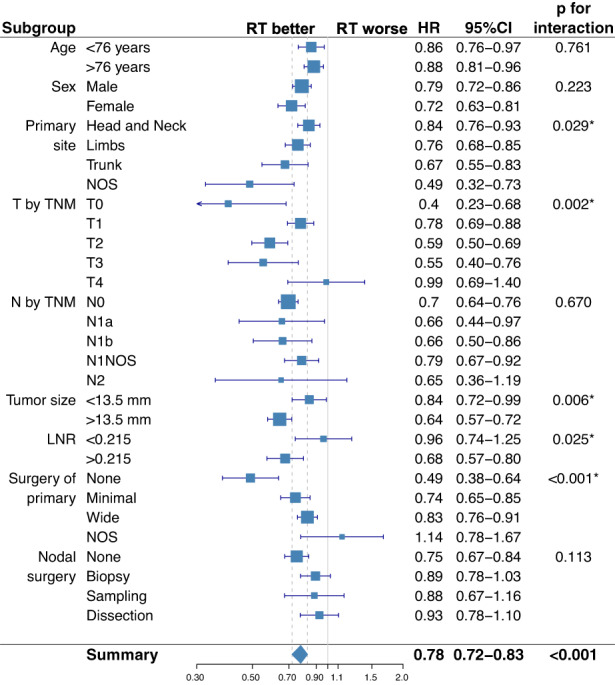
Subgroup analysis of the survival impact of radiation therapy. Forest plot summarizing subgroup analysis of the overall cohort of patients with nonmetastatic MCC (M0 MCC). RT, radiation therapy; 95% CI, 95% confidence interval; HR, hazard ratio; LNR, lymph node ratio; NOS, not otherwise specified

After correcting for potential confounding factors, RT was associated with a 25% reduction in the risk of death (HR: 0.75 [95% CI 0.68–0.82]; *p* < 0.001, Table 2). Subgroup analysis showed that OS benefit of RT was greater in MCC of unknown primary site (*p* = 0.029), T0 (*p* = 0.002), >13.5 mm in size (*p* = 0.006), LNR >0.215 (*p* = 0.025), and in patients who had not undergone resection of primary (*p* < 0.001). As opposed to this, RT did not significantly affect the outcome in T4 MCC, which probably reflects the presence of occult metastatic disease or the relatively small sample size (Figure 1).

**TABLE 2:** Univariate and multivariate Cox proportional hazard models for the risk of death in nonmetastatic Merkel cell carcinoma (M0 MCC) patients

Factor		Univariate			Multivariate		
		HR	95%CI	*p*	HR	95%CI	*p*
Sex	Male	1.37	1.28–1.48	**<0.001**	1.51	1.38–1.66	**<0.001**
Age	>76.5 years	2.66	2.47–2.85	**<0.001**	2.43	2.19–2.69	**<0.001**
Primary site	Limbs	1			1		
	Trunk	1.35	1.20–1.52	**<0.001**	0.85	0.70‐1.04	0.116
	Head&Neck	1.43	1.33–1.55	**<0.001**	0.97	0.79‐1.19	0.769
	NOS	1.13	0.91–1.39	0.263	1.18	0.64‐2.20	0.594
T by TNM	T0	1			–	–	–
	T1	1.01	0.78–1.32	0.928	1		
	T2	1.40	1.07–1.83	**0.014**	1.07	0.94–1.21	0.310
	T3	1.61	1.19–2.18	**0.002**	1.24	1.02–1.50	**0.029**
	T4	2.00	1.48–2.72	**<0.001**	1.43	1.16–1.76	**0.001**
Tumor size	>13.5 mm	1.57	1.42–1.72	**<0.001**	1.34	1.19–1.51	**<0.001**
N by TNM	N0	1			1		
	N1a	1.09	0.90–1.33	0.379	1.55	1.23–1.95	**<0.001**
	N1b	1.81	1.58–2.10	**<0.001**	2.79	2.34–3.23	**<0.001**
	N1 NOS	1.39	1.27–1.52	**<0.001**	1.95	1.68–2.72	**<0.001**
	N2	1.98	1.46–2.67	**<0.001**	2.24	1.57–3.19	**<0.001**
LNR	>0.215	1.58	1.32–1.88	**<0.001**	–	–	–
Surgery of primary	None	1			1		
	Minimal	0.88	0.77–1.01	0.065	0.97	0.79–1.19	0.769
	Wide	0.60	0.53–0.68	**<0.001**	0.85	0.70–1.04	0.116
	NOS	0.76	0.61–0.95	**0.017**	1.18	0.64–2.20	0.594
Nodal surgery	None	1			1		
	Biopsy	0.47	0.43–0.52	**<0.001**	0.53	0.46–0.59	**<0.001**
	Sampling	0.59	0.51–0.69	**<0.001**	0.52	0.43–0.63	**<0.001**
	Dissection	0.69	0.62–0.76	**<0.001**	0.61	0.53–0.72	**<0.001**
Radiation therapy	Yes	0.79	0.72–0.84	**<0.001**	0.75	0.68–0.82	**<0.001**

*Note*: Significant *p* values are highlighted in bold.

Abbreviations: CI, Confi.; HR, Hazard ratio

Since nodal involvement is known to be a key prognostic factor in MCC,^22^, ^23^ we also analyzed patients either with no nodal involvement (N0 MCC cohort) or with any degree of regional node involvement (N+ MCC cohort).

### 3.3 Impact of RT on survival in the N0 MCC cohort

First, we investigated the effect of RT in patients with an N0 MCC (*N* = 4022) by selecting those with no nodal involvement at diagnosis. Among patients in the N0 MCC cohort, 1637 (41%) received perioperative RT. Median OS in this cohort was 67 months (95% CI 62–72) and the 5‐year survival rate was 53%. Comparisons of survival by key prognostic groups in N0 MCC patients are reported in Table S3 and Figure S5. In addition, median OS was significantly longer in patients who received perioperative RT compared with those who did not (89 months [95% CI 79–99] vs 55.0 months [95% CI 50–60], respectively; *p* < 0.001, Figure S4B).

After correcting for potential confounding factors, RT was associated with a 27% reduction in the risk of death (HR: 0.73 [95% CI 0.65–0.82]; *p* < 0.001, Table 3).

**TABLE 3:** Univariate and multivariate Cox proportional hazard models for the risk of death in node‐negative Merkel cell carcinoma (N0 MCC) patients

Factor		Univariate			Multivariate		
		HR	95% CI	*p*	HR	95% CI	*p*
Sex	Male	1.44	1.32–1.58	**<0.001**	1.69	1.51–1.90	**<0.001**
Age	>76.5 years	3.22	2.92–3.55	**<0.001**	2.76	2.42–3.15	**<0.001**
Primary site	Limbs	1			1		
	Trunk	1.48	1.27–1.73	**<0.001**	1.39	1.14–1.69	**0.001**
	Head and Neck	1.59	1.45–1.74	**<0.001**	1.18	1.04–1.34	**0.008**
	NOS	2.16	1.30–3.60	**0.003**	1.38	0.71–2.67	0.342
T by TNM	T1	1			1		
	T2	1.37	1.21–1.55	**<0.001**			NS
	T3	1.31	1.02–1.69	**0.032**			NS
	T4	1.81	1.41–2.33	**<0.001**			NS
Tumor size	>13.5 mm	1.51	1.35–1.69	**<0.001**	1.52	1.35–1.70	**<0.001**
Surgery of primary	None	1			1		
	Minimal	0.70	0.58–0.84	**<0.001**			NS
	Wide	0.45	0.38–0.54	**<0.001**			NS
	NOS	0.52	0.36–0.75	**0.001**			NS
Nodal surgery	None	1			1		
	Biopsy	0.37	0.33–0.41	**<0.001**	0.49	0.42–0.57	**<0.001**
	Sampling	0.41	0.33–0.52	**<0.001**	0.50	0.39–0.65	**<0.001**
	Dissection	0.43	0.36–0.51	**<0.001**	0.58	0.47–0.73	**<0.001**
Radiation therapy	Yes	0.69	0.63–0.76	**<0.001**	0.73	0.65–0.82	**<0.001**

*Note*: Significant *p* values are highlighted in bold

Abbreviations: CI, Confidence interval; HR, Hazard ratio; NS, not significant.

**FIGURE 2:**
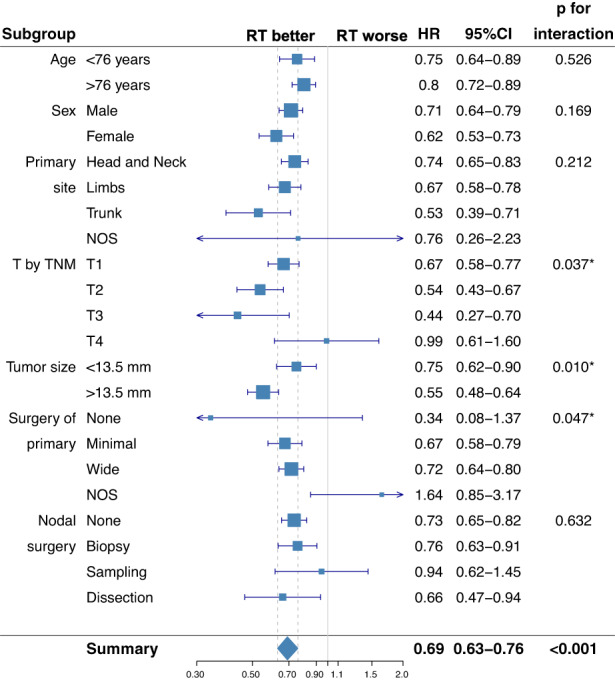
Subgroup analysis of the survival impact of radiation therapy. Forest plot summarizing subgroup analysis of the cohort of patients with node‐negative MCC (N0 MCC). RT, radiation therapy; 95% CI, 95% confidence interval; HR, hazard ratio; NOS, not otherwise specified

Subgroup analysis showed that RT was associated with a greater OS benefit in patients whose primary tumor was >13.5 mm in size (*p* = 0.01) and in those who had not undergone resection of primary (*p* = 0.047). On the contrary, RT did not significantly affect OS in T4 MCC similar to what was observed in the overall M0 MCC group (Figure 2).

### 3.4 Impact of RT on survival in the N+ MCC cohort

We then explored the efficacy of RT in patients with an MCC with regional lymph node involvement. To this end, we selected all patients with any type of nodal disease (N1a, N1b, N1‐NOS, *N* = 1551) and excluded those with in‐transit metastases (N2, *N* = 71).

Among patients in the N+ MCC cohort, perioperative RT was performed in 998 patients (64%). The median OS of patients in the N+ MCC cohort was 33 months (95% CI 29–37), and 5‐year OS was 38%. Comparisons of survival by key prognostic groups are reported in Table S4 and Figure S6. Furthermore, median OS was longer in patients who received perioperative RT compared with those who did not (41 months [95% CI 34–48] vs 25 months [95% CI 20–30], respectively; *p* < 0.001, Figure S4C).

After correcting for potential confounding factors, an LNR >0.215 retained its association with an increased risk of death (HR: 1.78 [95% CI 1.41–2.26]; *p* < 0.001), and RT was associated with a 26% reduction of the risk of death (HR: 0.74 [95% CI 0.60–0.90]; *p* = 0.003, Table 4).

**TABLE 4:** Univariate and multivariate Cox proportional hazard models for the risk of death in node‐positive Merkel cell carcinoma (N+ MCC) patients. A multivariate model was fitted on N = 735 cases with available data for all considered factors

Factor		Univariate			Multivariate		
		HR	95% CI	*p*	HR	95% CI	*p*
Sex	Male	1.13	0.98–1.29	0.075	–	–	–
Age	>76.5 years	2.19	1.92–2.48	**<0.001**	1.90	1.57–2.31	**<0.001**
Primary site	Limbs	1			1		
	Trunk	1.04	0.86–1.26	0.671			NS
	Head and Neck	1.21	1.05–1.40	**0.008**			NS
	NOS	0.69	0.54–0.88	**0.003**			NS
Tumor size	>13.5 mm	1.39	1.14–1.70	**0.001**	1.21	0.97–1.51	0.09
N by TNM	N1a	1			1		
	N1b	1.65	1.31–2.08	**<0.001**	1.47	1.10–1.96	**0.009**
	N1 NOS	1.39	1.12–1.71	**0.002**	1.27	0.99–1.64	0.063
LNR	>0.215	1.51	1.30–1.76	**<0.001**	1.78	1.41–2.26	**<0.001**
Nodal surgery	None	1			1		
	Biopsy	0.56	0.45–0.71	**<0.001**	0.68	0.25–1.86	0.455
	Sampling	0.55	0.42–0.72	**<0.001**	0.78	0.28–2.15	0.632
	Dissection	0.56	0.45–0.69	**<0.001**	1.11	0.41–3.02	0.833
Radiation therapy	Yes	0.75	0.66–0.85	**<0.001**	0.74	0.60–0.90	**0.003**

*Note*: Significant *p* values are highlighted in bold

Abbreviations: CI, Confidence interval; HR, Hazard ratio; LNR, lymph node ratio; NS, not significant.

**FIGURE 3:**
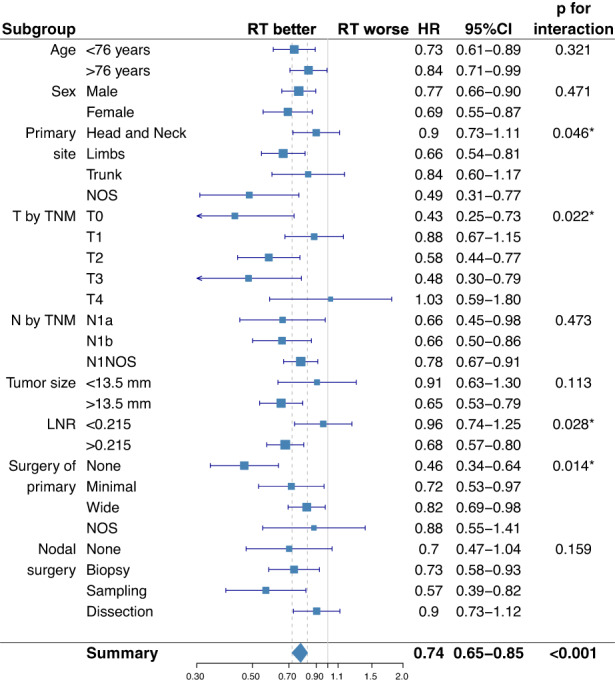
Subgroup analysis of the survival impact of radiation therapy. Forest plot summarizing subgroup analysis of the cohort of patients with node‐positive MCC (N+ MCC). RT, radiation therapy; 95% CI, 95% confidence interval; HR, hazard ratio; LNR, lymph node ratio; NOS, not otherwise specified

Subgroup analysis showed that RT yielded greater OS benefit in patients with MCC of unknown primary site (*p* = 0.046), T0 (*p* = 0.022), LNR >0.215 (*p* = 0.028), and in patients who had not undergone resection of primary (*p* = 0.014). As opposed to this, RT did not significantly affect the outcome in T4 MCC, which again probably reflects the presence of occult metastatic disease or the small sample size of this subgroup (Figure 3).

## 4 DISCUSSION

Our population‐based analysis showed that RT was associated with improved OS in M0 MCC, irrespective of the nodal status and other potential confounding factors. Furthermore, benefit from RT was increased in tumors >13.5 mm in size (N0 MCC cohort), whereas less benefit was derived from RT in T4 MCC (in both N0 and N+ cohorts). Notably, an LNR >0.215 identified a subgroup of patients with MCC with worse prognosis within the N+ MCC cohort that derives the most benefit from perioperative RT.

Adjuvant RT on primary tumor bed and draining nodal basin, which is recommended after resection of a nonmetastatic MCC, is associated with a reduction in local relapse rate, while adjuvant RT association with improvement in OS is uncertain.^8^, ^9^

Indeed, RT also improves survival in MCC with no nodal involvement.^17^, ^18^, ^19^ On the other hand, OS is improved by adjuvant RT in MCC with nodal involvement (N+, i.e., stage III) according to some reports,^10^, ^19^ while many others,^11^, ^12^, ^13^, ^14^, ^17^ including one small randomized clinical trial,^16^ did not highlight a survival advantage in patients receiving RT compared with those who did not receive it. The lack of definitive data about OS improvement by RT may be due to incomplete risk stratification of N+ MCC patients, also because some risk factors are not captured by retrospective databases or by registries.

LNR is a recognized prognostic factor in cancer,^24^, ^25^ likely linked to its association with the probability of residual disease after surgery,^26^, ^27^, ^28^ and can also aid decision‐making in the oral cavity, cervical, and non‐small‐cell lung cancer.^29^, ^30^, ^31^ In a recently published population‐based study in N+ MCC patients, an LNR >0.31 was associated with worse OS and identified patients who derived increased survival benefits from adjuvant chemo‐RT compared with RT alone or no adjuvant therapy after surgery.^32^ In the same study, no difference was observed between adjuvant chemo‐RT and other postoperative approaches in MCC patients with LNR <0.31. Nevertheless, the LNR cutoff was arbitrarily chosen as the highest quartile of LNR values, thus introducing a potential bias. In this analysis, the LNR which best‐identified patients with worse prognosis was calculated in an unbiased way by the ROC curve, and an increased OS benefit from RT in patients with an LNR >0.215 compared to those with an LNR <0.215 was observed.

These findings may help interpret results and stratify patients in clinical trials of perioperative management of MCC patients, especially those of RT combined with other treatments such as immune checkpoint inhibitors. Similarly, RT had no impact on survival in T4 MCC likely because this group included patients with occult systemic disease in which local treatment have a limited role. On the contrary, T0 or unknown primary site MCC is associated with increased survival benefits from RT in the overall cohort and the N+ MCC cohort. The improved effect of RT in these patients can be explained by the fact that radiation fields can effectively encompass all the residual disease in the nodal basin or because of an immune reaction toward the MCC that is amplified by RT. Indeed, up to 10% of MCCs have no evident primary tumor or present primary tumor spontaneous regression and show improved survival compared to matched MCC with present primary tumor^33^, ^34^, ^35^ possibly due to an immunological response against tumor cells, which can be intensified by RT.^36^

Limitations of our study include the lack of potentially relevant confounding factors, mainly performance status, type and doses of RT, and adjuvant and subsequent treatments. However, the use of registries is of paramount importance to analyze a meaningful number of patients with rare diseases such as MCC. With respect to lacking data about adjuvant treatments, it has to be considered that chemotherapy did not prove to affect survival,^21^ while the newly investigated immune checkpoint inhibitors are not likely to overall affect the results of the present study given the small proportion of patients who could have had access to these treatments before 2016. In addition, we used OS as the primary endpoint, as opposite to cancer‐specific survival, to capture also toxic detrimental effects of treatment in these patients, as they are often old, comorbid, and thus frail.

In conclusion, our findings suggest that RT improves survival in M0 MCC, irrespective of nodal status, but that it does not impact OS in patients with T4 tumors. An LNR cutoff of 0.215 in N+ MCC is a useful prognostic factor for decision‐making and design and interpretation of clinical trials in nonmetastatic MCC.

## AUTHORS CONTRIBUTION

GL and DC conceptualization, data analysis, and writing of the first draft; GL, EA, PVM, and GR data collection; EA, GS, CM, VA, CR, PVM, GR, and RC critical review of the manuscript; DC project supervision; all authors approval of the final version.

## ETHICS STATEMENT

Permission to access the SEER database was granted on 19/03/2020 with authorization number 21495‐Nov2018.

## CONFLICT OF INTEREST

The authors have no conflict of interest to declare.

## Supporting information


Fig S1

Fig S2

Fig S3

Fig S4

Fig S5

Fig S6

Table S1

Table S2

Table S3

Table S4

## Data Availability

Data sharing is not applicable to this article as no new data were created or analyzed in this study.
