# Supplementary material for: Lymph node ratio predicts efficacy of postoperative radiation therapy in nonmetastatic Merkel cell carcinoma: A population‐based analysis
Source: Cancer Med. 2022 Apr 29;11(22):4204–13. doi: 10.1002/cam4.4773 (PMC9678092; doi:10.1002/cam4.4773)

Supplementary Figure 1. Study flow-chart. Flow-chart of Merkel cell carcinoma (MCC) patients extracted from SEER registry and included in the study. Boxes with bold edge identify cohorts analyzed in the study: black-edged box is the overall M0 MCC cohort, blue-edged box is the node-negative (N0) cohort and red-edged cohort is the node-positive (N+) cohort.

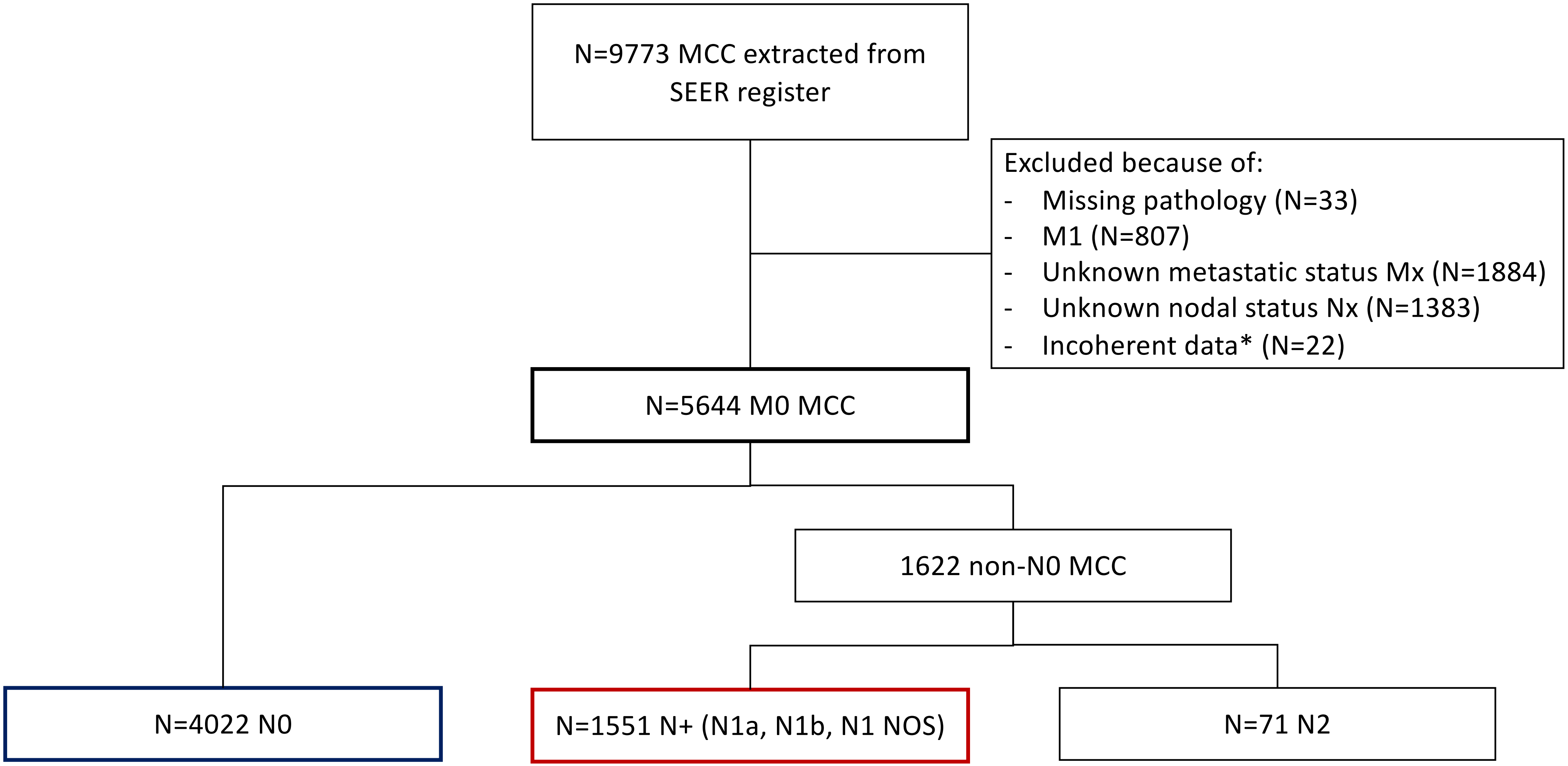

Supplement: Supplementary file 1 — Fig S1 [file CAM4-11-4204-s006.pdf]
