# Supplementary material for: Lymph node ratio predicts efficacy of postoperative radiation therapy in nonmetastatic Merkel cell carcinoma: A population‐based analysis
Source: Cancer Med. 2022 Apr 29;11(22):4204–13. doi: 10.1002/cam4.4773 (PMC9678092; doi:10.1002/cam4.4773)
Supplement: Supplementary file 2 — Fig S2 [file CAM4-11-4204-s002.pdf]

Supplementary Figure 2. Receiving-Operator Characteristics (ROC) curve used to determine the best cut-off of (A) the age, (B) the tumor size in millimeters (B), and (C) the lymph node ratio (ratio of positive lymph nodes at pathology out of the number of analyzed lymph nodes, LNR) and their accuracy in predicting 5-year overall survival. * identifies the best-cut-off on the curve.

A

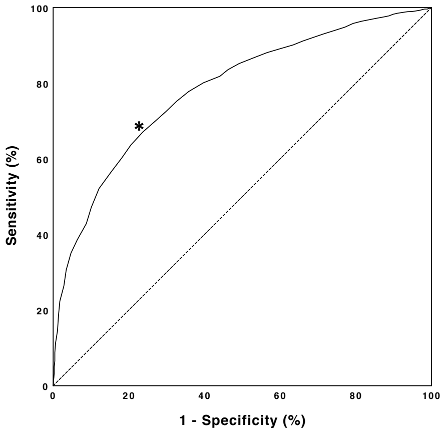

B

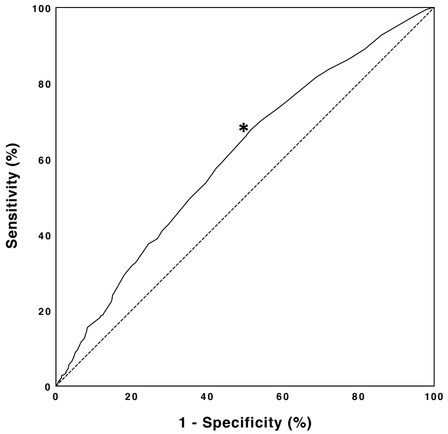

C

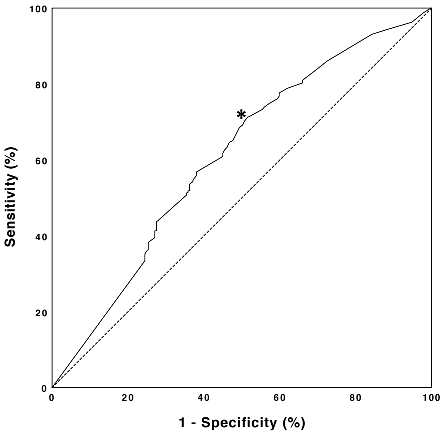

**Variable**	***Age***	***Tumor size***	***LNR***
**Cut-off**	76.5 years	13.5 mm	0.215
**AUC**	0.784	0.602	0.609
**Sensitivity**	67%	67%	71%
**Specificity**	76%	49%	48%
p	<0.001	<0.001	<0.001
