# Supplementary material for: Lymph node ratio predicts efficacy of postoperative radiation therapy in nonmetastatic Merkel cell carcinoma: A population‐based analysis
Source: Cancer Med. 2022 Apr 29;11(22):4204–13. doi: 10.1002/cam4.4773 (PMC9678092; doi:10.1002/cam4.4773)

Supplementary Figure 3. Kaplan-Meier estimates of overall survival according to (A) sex, (B) age, (C) primary site, (D) stage at diagnosis according to AJCC 7° edition, (E) T parameter according to TNM, (F) primary tumor size, (G) Lymph node ratio (LNR), (H) N parameter according to TNM, (I) surgery of primary, and (J) node-directed surgery.

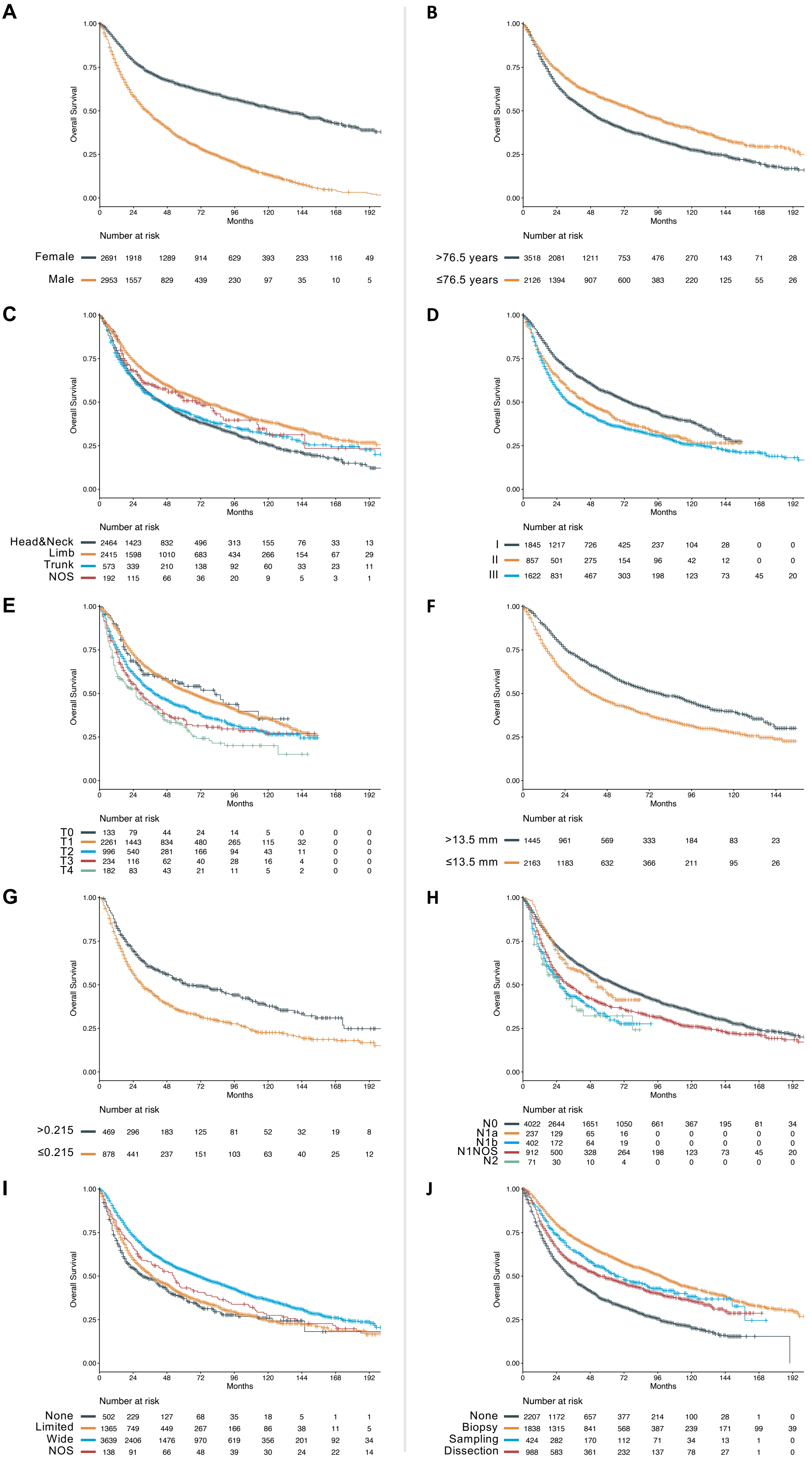

Supplement: Supplementary file 3 — Fig S3 [file CAM4-11-4204-s010.pdf]
