# Supplementary material for: Lymph node ratio predicts efficacy of postoperative radiation therapy in nonmetastatic Merkel cell carcinoma: A population‐based analysis
Source: Cancer Med. 2022 Apr 29;11(22):4204–13. doi: 10.1002/cam4.4773 (PMC9678092; doi:10.1002/cam4.4773)

Supplementary Figure 4. Kaplan-Meier estimates of overall survival in (A) non-metastatic Merkel cell carcinoma (M0 MCC), (B) node-negative MCC (N0 MCC), and (C) node-positive MCC (N+ MCC). *p*-value is for log-rank test.

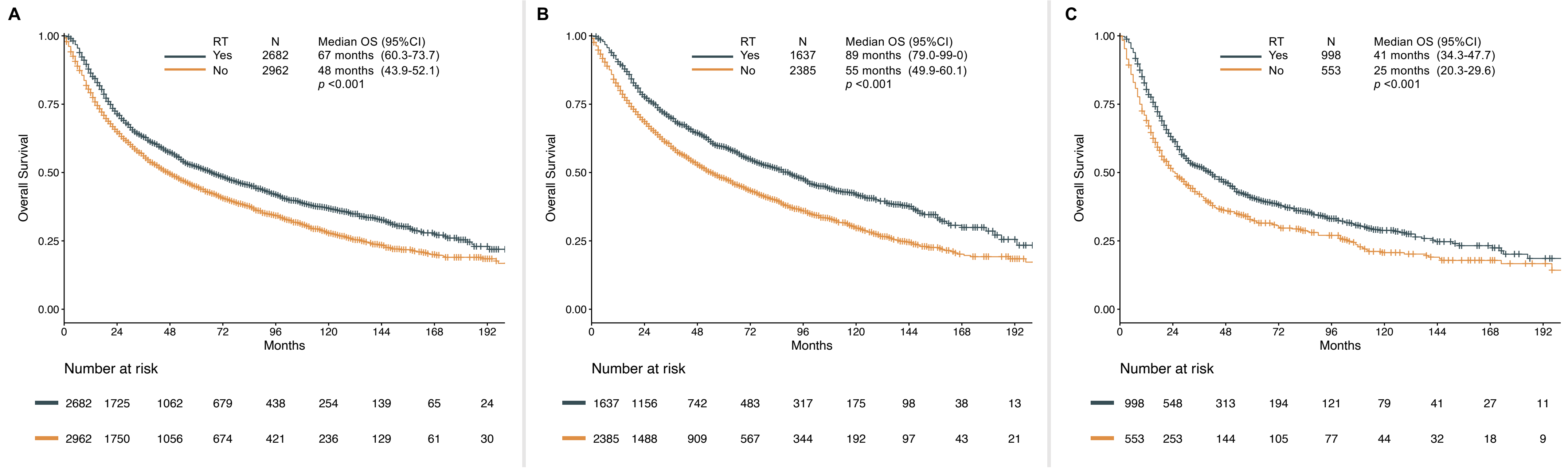

Supplement: Supplementary file 4 — Fig S4 [file CAM4-11-4204-s009.pdf]
