# Supplementary material for: Lymph node ratio predicts efficacy of postoperative radiation therapy in nonmetastatic Merkel cell carcinoma: A population‐based analysis
Source: Cancer Med. 2022 Apr 29;11(22):4204–13. doi: 10.1002/cam4.4773 (PMC9678092; doi:10.1002/cam4.4773)

Supplementary Figure 4. Kaplan-Meier estimates of overall survival according to (A) sex, (B) age, (C) primary site, (D) T parameter according to TNM, (E) primary tumor size, (F) surgery of primary, and (G) node-directed surgery.

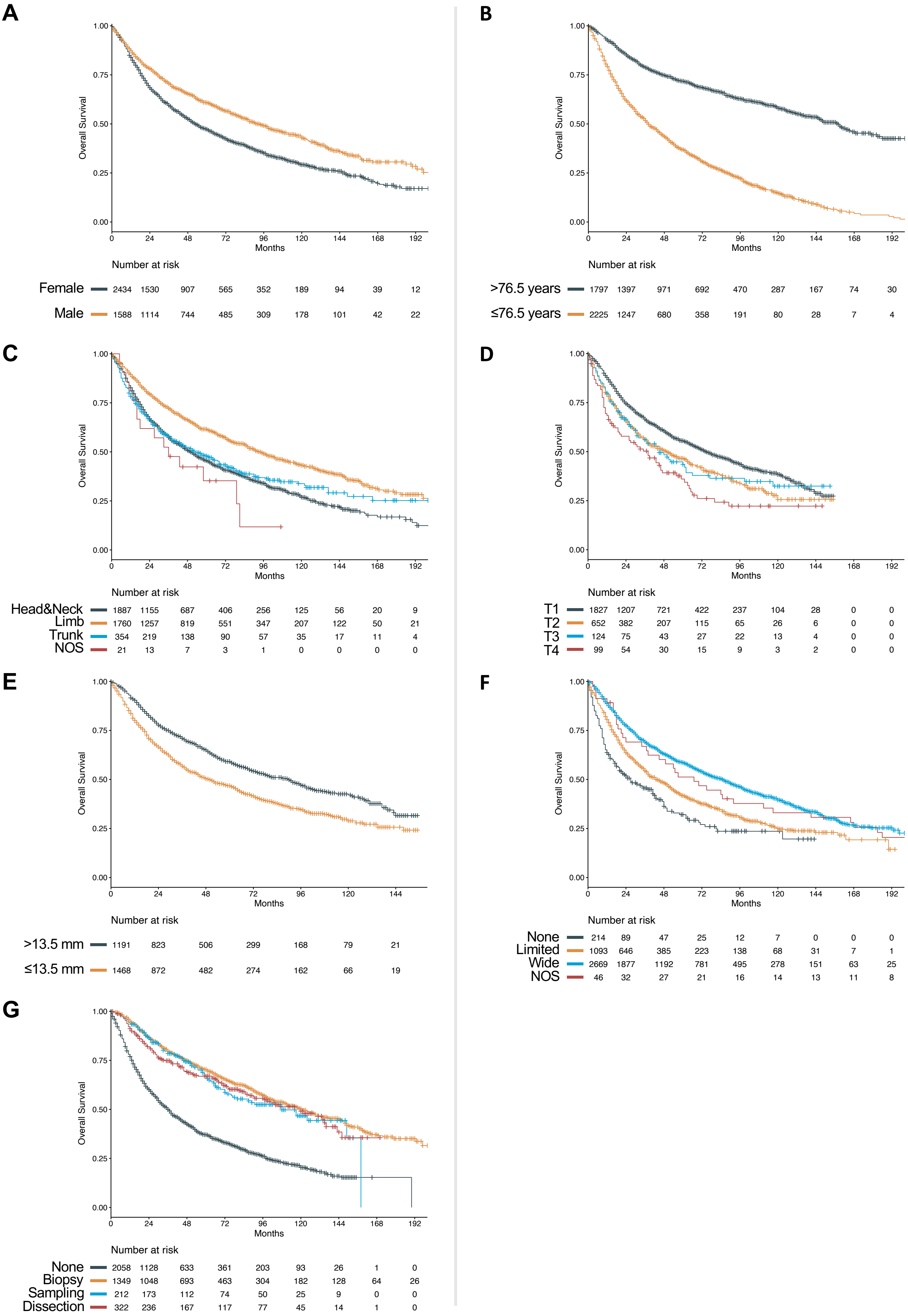

Supplement: Supplementary file 5 — Fig S5 [file CAM4-11-4204-s005.pdf]
