# Supplementary material for: Lymph node ratio predicts efficacy of postoperative radiation therapy in nonmetastatic Merkel cell carcinoma: A population‐based analysis
Source: Cancer Med. 2022 Apr 29;11(22):4204–13. doi: 10.1002/cam4.4773 (PMC9678092; doi:10.1002/cam4.4773)

Supplementary Figure 5. Kaplan-Meier estimates of overall survival according to (A) sex, (B) age, (C) primary site, (D) T parameter according to TNM, (E) primary tumor size, (F) Lymph node ratio (LNR), (G) N parameter according to TNM, (H) surgery of primary, and (I) node-directed surgery.

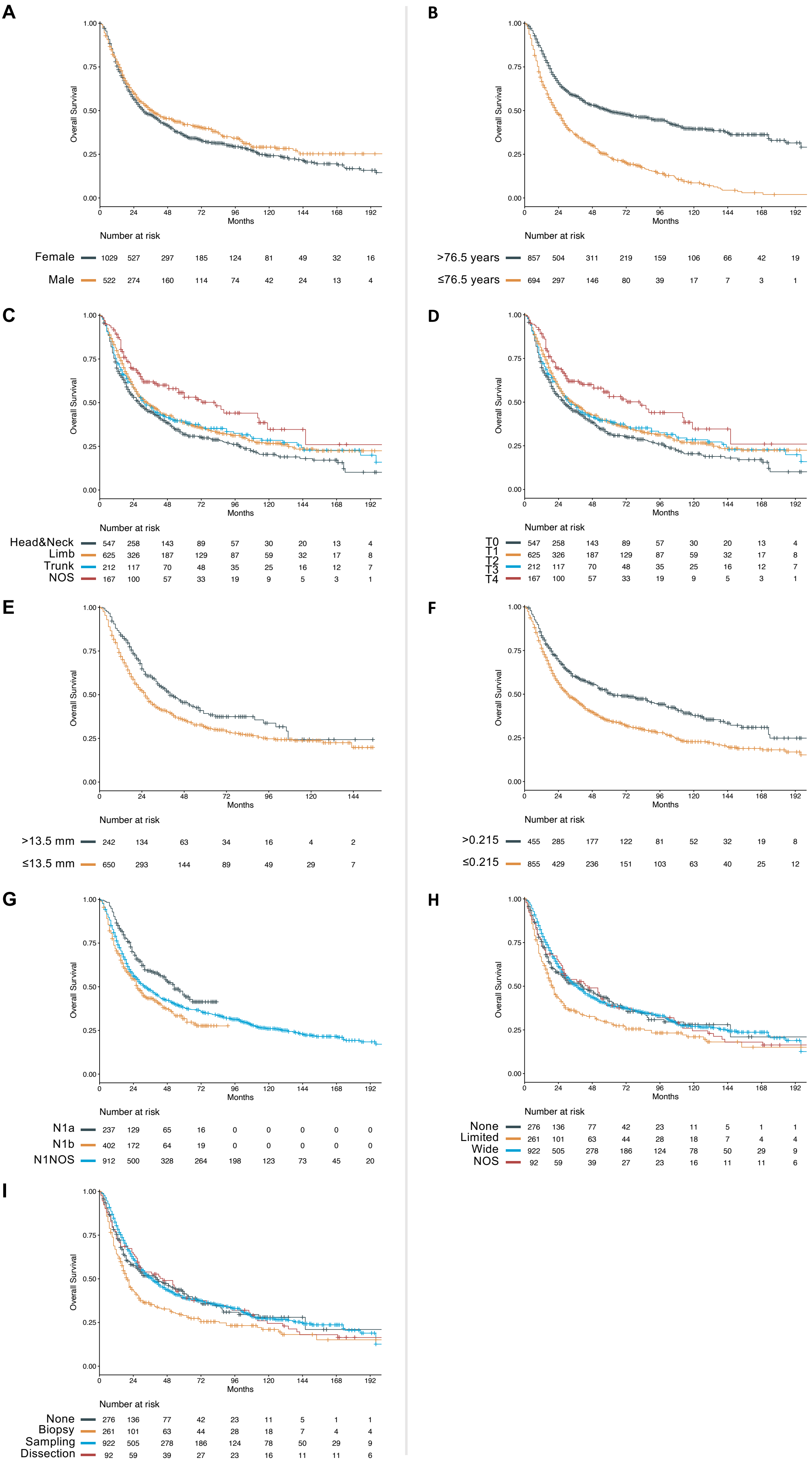

Supplement: Supplementary file 6 — Fig S6 [file CAM4-11-4204-s004.pdf]
