# Supplementary material for: Lymph node ratio predicts efficacy of postoperative radiation therapy in nonmetastatic Merkel cell carcinoma: A population‐based analysis
Source: Cancer Med. 2022 Apr 29;11(22):4204–13. doi: 10.1002/cam4.4773 (PMC9678092; doi:10.1002/cam4.4773)
Supplement: Supplementary file 7 — Table S1 [file CAM4-11-4204-s001.docx]

**Supplementary Table 2.** Imbalances in radiation therapy (RT) delivery according to key prognostic factors in Merkel cell carcinoma (MCC).

| **Variable** | | **M0 MCC** | | | **N0 MCC** | | | **N+ MCC** | | |
| --- | --- | --- | --- | --- | --- | --- | --- | --- | --- | --- |
|  |  | *RT* | *No RT* | *p* | *RT* | *No RT* | *p* | *RT* | *No RT* | *p* |
| **Age** | **≤76.5 years** | 1480 (55.0%) | 1211 (45.0%) | ***<0.001*** | 869 (48.4%) | 928 (51.6%) | ***<0.001*** | 586 (68.4%) | 271 (31.6%) | ***<0.001*** |
|  | **>76.5 years** | 1202 (40.7%) | 1751 (59.3%) |  | 768 (34.5%) | 1457 (65.5%) |  | 412 (59.4%) | 282 (40.6%) |  |
| **Sex** | **Male** | 1748 (49.7%) | 1770 (50.3%) | ***<0.001*** | 1029 (42.3%) | 1405 (57.7%) | ***0.012*** | 680 (66.1%) | 349 (33.9%) | ***0.045*** |
|  | **Female** | 934 (43.9%) | 1192 (56.1%) |  | 608 (38.3%) | 980 (61.7%) |  | 318 (60.9%) | 204 (39.1%) |  |
| **Primary site** | **Head&Neck** | 1149 (46.6%) | 1315 (53.4%) | ***<0.001*** | 762  (40.4%) | 1125  (59.6%) | 0.947 | 364 (66.5%) | 183 (33.5%) | 0.154 |
|  | **Limbs** | 1130 (46.8%) | 1285 (53.2%) |  | 725 (41.2%) | 1035  (58.8%) |  | 387 (61.9%) | 238 (38.1%) |  |
|  | **Trunk** | 276 (48.2%) | 297 (51.8%) |  | 142 (40.1%) | 212 (59.9%) |  | 131 (61.8%) | 81 (38.2%) |  |
|  | **NOS** | 127 (66.1%) | 65 (33.9%) |  | 8  (38.1%) | 13 (61.9%) |  | 116 (69.5%) | 51 (30.5%) |  |
| **Stage at diagnosis** | **I** | 773 (41.9%) | 1072 (58.1%) | ***<0.001*** | 773 (41.9%) | 1072 (58.1%) | ***<0.001*** | - | - | - |
|  | **II** | 421 (49.1%) | 436 (50.9%) |  | 421 (49.1%) | 436 (50.9%) |  | - | - |  |
|  | **III** | 1045 (64.4%) | 577 (35.6%) |  | - | - |  | - | - |  |
| **T by TNM** | **T0** | 99  (74.4%) | 34  (25.6%) | ***<0.001*** | - | - | ***0.002*** | 97  (74.6%) | 33  (25.4%) | 0.404 |
|  | **T1** | 1063  (47.0%) | 1198  (53.0%) |  | 763  (41.8%) | 1064  (58.2%) |  | 285  (68.5%) | 131  (31.5%) |  |
|  | **T2** | 544  (54.6%) | 452  (45.4%) |  | 314  (48.2%) | 338  (51.8%) |  | 212  (67.1%) | 104  (32.9%) |  |
|  | **T3** | 136  (58.1) | 98  (41.9%) |  | 66  (53.2%) | 58  (46.8%) |  | 63  (65.6%) | 33  (34.4%) |  |
|  | **T4** | 103  (56.6%) | 79  (43.4%) |  | 51  (51.5%) | 48  (48.5%) |  | 47  (62.7%) | 28  (37.3%) |  |
| **Tumor size** | **≤13.5 mm** | 652 (45.1%) | 561 (61.5%) | ***<0.001*** | 485 (40.7%) | 706 (59.3%) | ***0.001*** | 157 (64.9%) | 85 (35.1%) | 0.401 |
|  | **>13.5 mm** | 1161 (53.7%) | 1002 (46.3%) |  | 692 (47.1%) | 776 (52.9%) |  | 441 (67.8%) | 209 (32.2%) |  |
| **N by TNM** | **N0** | 1637 (40.7%) | 2385 (59.3%) | ***<0.001*** | - | - |  | - | - | ***0.016*** |
|  | **N1a** | 158 (66.7%) | 79 (33.3%) |  | - | - |  | 158 (66.7%) | 79 (33.3%) |  |
|  | **N1b** | 279 (69.4%) | 123 (30.6%) |  | - | - |  | 279 (69.4%) | 123 (30.6%) |  |
|  | **N1 NOS** | 561 (61.5%) | 351 (38.5%) |  | - | - |  | 561 (61.5%) | 351 (38.5%) |  |
|  | **N2** | 47 (66.2%) | 24 (33.8%) |  | - | - |  | - | - |  |
| **LNR** | **≤0.215** | 300  (64.0%) | 169  (36.0%) | 0.201 | - | - | - | 291  (64.0%) | 164  (36.0%) | 0.247 |
|  | **>0.215** | 592  (67.4%) | 286  (32.6%) |  | - | - |  | 574  (67.1%) | 281  (32.9%) |  |
| **Surgery of primary** | **None** | 169  (33.7%) | 333 (66.3%) | ***<0.001*** | 6  (2.8%) | 208  (97.2%) | ***<0.001*** | 160  (58.0%) | 116  (42.0%) | ***0.031*** |
|  | **Minimal** | 673  (49.3%) | 692 (50.7%) |  | 485  (44.4%) | 608  (55.6%) |  | 179  (68.6%) | 82  (31.4%) |  |
|  | **Wide** | 1767  (48.6%) | 1872 (51.4%) |  | 1127  (42.2%) | 1542  (57.8%) |  | 605  (65.6%) | 317  (34.4%) |  |
|  | **NOS** | 73  (52.9%) | 65  (47.1%) |  | 19  (41.3%) | 27  (58.7%) |  | 54  (58.7%) | 38  (41.3%) |  |
| **Nodal surgery** | **None** | 773 (35.0%) | 1434 (65.0%) | ***<0.001*** | 706 (34.3%) | 1352 (65.7%) | ***<0.001*** | 59 (45.0%) | 72 (55.0%) | ***<0.001*** |
|  | **Biopsy** | 973 (52.9%) | 865 (47.1%) |  | 635 (47.1%) | 714 (52.9%) |  | 324 (68.9%) | 146 (31.1%) |  |
|  | **Sampling** | 245 (57.8%) | 179 (42.2%) |  | 100 (47.2%) | 112 (52.8%) |  | 136 (67.3%) | 66 (32.7%) |  |
|  | **Dissection** | 607 (61.4%) | 381 (38.6%) |  | 166 (51.6%) | 156 (48.4%) |  | 426 (66.1%) | 218 (33.9%) |  |

M0 MCC: non-metastatic Merkel cell carcinoma; N0 MCC: node-negative Merkel cell carcinoma; N+ MCC: node-positive Merkel cell carcinoma; LNR: lymph node ratio; NA: not available.
