# Supplementary material for: Lymph node ratio predicts efficacy of postoperative radiation therapy in nonmetastatic Merkel cell carcinoma: A population‐based analysis
Source: Cancer Med. 2022 Apr 29;11(22):4204–13. doi: 10.1002/cam4.4773 (PMC9678092; doi:10.1002/cam4.4773)
Supplement: Supplementary file 8 — Table S2 [file CAM4-11-4204-s003.docx]

**Supplementary Table 3.** Comparison by log-rank test of median overall survival by key prognostic factors. Median expressed in months.

| **Variable** | **Groups** | **Median** | **95%CI** | **p** |
| --- | --- | --- | --- | --- |
| **Age** | ≤76.5 years | 131 | (117.3-144.7) | <0.001 |
|  | >76.5 years | 34 | (31.7-36.3) |  |
| **Sex** | Female | 81 | (72.9-89.1) | <0.001 |
|  | Male | 46 | (42.3-49.2) |  |
| **Primary site** | Limb | 75 | (67.4-82.5) | <0.001 |
|  | Head&Neck | 45 | (41.2-48.8) |  |
|  | Trunk | 44 | (33.4-54.6) |  |
|  | NOS | 69 | (47.1-90.8) |  |
| **Stage at diagnosis** | I | 75 | (66.9-83.1) | <0.001 |
|  | II | 45 | (37.5-52.5) |  |
|  | III | 32 | (28.2-35.8) |  |
| **T by TNM** | T0 | 82 | (53.0-111.0) | <0.001 |
|  | T1 | 66 | (59.0-72.9) |  |
|  | T2 | 39 | (32.8-45.2) |  |
|  | T3 | 31 | (23.6-38.4) |  |
|  | T4 | 26 | (16.2-35.8) |  |
| **Tumor size** | ≤13.5 mm | 78 | (67.4-88.6) | <0.001 |
|  | >13.5 mm | 39 | (35.0-43.0) |  |
| **N by TNM** | N0 | 67 | (62.1-71.4) | <0.001 |
|  | N1a | 53 | (42.2-63.8) |  |
|  | N1b | 27 | (22.9-31.1) |  |
|  | N1NOS | 32 | (27.2-36.8) |  |
|  | N2 | 26 | (14.3-37.7) |  |
| **LNR*** | ≤0.215 | 63 | (43.7-82.3) | <0.001 |
|  | >0.215 | 31 | (26.8-35.2) |  |
| **Surgery of primary** | None | 31 | (22.4-39.6) | <0.001 |
|  | Minimal | 38 | (32.8-43.2) |  |
|  | Wide | 71 | (64.9-77.1) |  |
|  | NOS | 53 | (41.6-64.4) |  |
| **Node-directed surgery** | None | 34 | (31.2-36.8) | <0.001 |
|  | Biopsy | 99 | (90.0-107.9) |  |
|  | Sampling | 67 | (50.0-84.0) |  |
|  | Dissection | 56 | (45.3-66.7) |  |

*calculated only in patients with at least 1 positive lymph node and available data (N=1347). CI: Confidence Interval.
