# Supplementary material for: Lymph node ratio predicts efficacy of postoperative radiation therapy in nonmetastatic Merkel cell carcinoma: A population‐based analysis
Source: Cancer Med. 2022 Apr 29;11(22):4204–13. doi: 10.1002/cam4.4773 (PMC9678092; doi:10.1002/cam4.4773)
Supplement: Supplementary file 9 — Table S3 [file CAM4-11-4204-s008.docx]

**Supplementary Table 4.** Comparison by log-rank test of median overall survival by key prognostic factors in the node-negative Merkel cell carcinoma group (N0 MCC). Median expressed in months.

| **Variable** | **Groups** | **Median** | **95%CI** | **p** |
| --- | --- | --- | --- | --- |
| **Age** | ≤76.5 years | 158 | (140.8-175.2) | <0.001 |
|  | >76.5 years | 38 | (34.8-41.1) |  |
| **Sex** | Female | 92 | (81.9-102.1) | <0.001 |
|  | Male | 53 | (48.4-57.5) |  |
| **Primary site** | Limb | 94 | (83.8-104.1) | <0.001 |
|  | Head&Neck | 50 | (44.7-55.3) |  |
|  | Trunk | 53 | (39.9-66.0) |  |
|  | NOS | 36 | (12.9-59.0) |  |
| **T by TNM** | T1 | 75 | (66.9-83.1) | <0.001 |
|  | T2 | 50 | (40.0-60.0) |  |
|  | T3 | 45 | (30.7-59.2) |  |
|  | T4 | 38 | (22.9-53.1) |  |
| **Tumor size** | ≤13.5 mm | 90 | (78.8-101.2) | <0.001 |
|  | >13.5 mm | 50 | (43.2-56.8) |  |
| **Surgery of primary** | None | 27 | (15.2-38.8) | <0.001 |
|  | Minimal | 43 | (37.6-48.4) |  |
|  | Wide | 86 | (79.4-92.6) |  |
|  | NOS | 67 | (39.5-94.5) |  |
| **Node-directed surgery** | None | 36 | (32.9-39.0) | <0.001 |
|  | Biopsy | 122 | (105.8-138.2) |  |
|  | Sampling | 108 | (73.7-142.3) |  |
|  | Dissection | 120 | (90.9-149.1) |  |

CI: Confidence Interval.
