# Supplementary material for: Lymph node ratio predicts efficacy of postoperative radiation therapy in nonmetastatic Merkel cell carcinoma: A population‐based analysis
Source: Cancer Med. 2022 Apr 29;11(22):4204–13. doi: 10.1002/cam4.4773 (PMC9678092; doi:10.1002/cam4.4773)
Supplement: Supplementary file 10 — Table S4 [file CAM4-11-4204-s007.docx]

**Supplementary Table 5.** Comparison by log-rank test of median overall survival by key prognostic factors in the node-positive Merkel cell carcinoma group (N+ MCC). Median expressed in months.

| **Variable** | **Groups** | **Median** | **95%CI** | **p** |
| --- | --- | --- | --- | --- |
| **Age** | ≤76.5 years | 60 | (43.2-76.8) | <0.001 |
|  | >76.5 years | 23 | (20.2-25.8) |  |
| **Sex** | Female | 37 | (27.1-46.9) | 0.072 |
|  | Male | 31 | (26.5-35.5) |  |
| **Primary site** | Limb | 34 | (28.2-39.8) | <0.001 |
|  | Head&Neck | 28 | (22.9-33.1) |  |
|  | Trunk | 31 | (22.7-39.3) |  |
|  | NOS | 82 | (55.8-108.2) |  |
| **T by TNM** | T0 | 82 | (53.0-111.0) | <0.001 |
|  | T1 | 38 | (30.0-46.0) |  |
|  | T2 | 28 | (21.7-34.3) |  |
|  | T3 | 19 | (13.3-24.7) |  |
|  | T4 | 18 | (7.1-28.9) |  |
| **Tumor size** | ≤13.5 mm | 40 | (29.1-50.1) | 0.001 |
|  | >13.5 mm | 26 | (22.8-29.2) |  |
| **N by TNM** | N1a | 53 | (42.2-63.8) | <0.001 |
|  | N1b | 27 | (22.9-31.1) |  |
|  | N1NOS | 32 | (27.2-36.9) |  |
| **LNR*** | ≤0.215 | 62 | (42.0-82.0) | <0.001 |
|  | >0.215 | 31 | (26.5-35.5) |  |
| **Surgery of primary** | None | 41 | (26.0-55.8) | <0.001 |
|  | Minimal | 20 | (16.9-23.1) |  |
|  | Wide | 38 | (33.0-43.0) |  |
|  | NOS | 45 | (24.6-65.4) |  |
| **Node-directed surgery** | None | 16 | (12.6-19.4) | <0.001 |
|  | Biopsy | 39 | (31.0-47.0) |  |
|  | Sampling | 34 | (23.8-44.2) |  |
|  | Dissection | 35 | (27.7-42.2) |  |

*calculated only in patients with at least 1 positive lymph node and available data (N=1310). CI: Confidence Interval.
